# Identification of human papillomaviruses from formalin-fixed, paraffin-embedded pre-cancer and invasive cervical cancer specimens in Zambia: a cross-sectional study

**DOI:** 10.1186/s12985-014-0234-8

**Published:** 2015-01-16

**Authors:** Allen C Bateman, Katundu Katundu, Pascal Polepole, Aaron Shibemba, Mulindi Mwanahamuntu, Dirk P Dittmer, Groesbeck P Parham, Carla J Chibwesha

**Affiliations:** Centre for Infectious Disease Research in Zambia, Plot 5032 Great North Road, Lusaka, Zambia; Department of Medicine, UNC School of Medicine, University of North Carolina at Chapel Hill, Chapel Hill, North Carolina USA; University of Zambia Teaching Hospital, Lusaka, Zambia; Program in Global Oncology, UNC Lineberger Comprehensive Cancer Center and School of Medicine, UNC, Chapel Hill, North Carolina USA; Department of Obstetrics and Gynecology, UNC School of Medicine, UNC, Chapel Hill, North Carolina USA

**Keywords:** Cervical cancer, Formalin-fixed paraffin-embedded, Human papillomavirus, Zambia

## Abstract

**Background:**

The most common human papillomavirus (HPV) genotypes isolated from cervical cancer in select African countries are HPV-16, HPV-18, HPV-35, and HPV-45, but the most common genotypes in Zambia are unknown. The overall objective of this study was to assess the potential impact of current HPV vaccines in preventing cervical cancer in Zambia, by determining the combined prevalence of HPV-16 and/or HPV-18 in invasive cervical cancer (ICC) and high-grade pre-cancer [cervical intraepithelial neoplasia 2 or 3 (CIN2/3)] cases.

**Findings:**

We compared DNA extraction techniques to determine which assay performs well in the Zambian context, where unbuffered formalin is used to fix specimens. We then tested specimens with the Abbott RealTime High-Risk HPV test to estimate the prevalence of HPV-16/18 in formalin-fixed, paraffin-embedded ICC and CIN2/3 specimens. DNA extraction using heat (without xylene) was more successful than xylene-based extraction. Over 80% of specimens tested using heat extraction and the Abbott RealTime HPV test were positive for HPV. HPV-16 and/or HPV-18 were identified in 65/93 (69.9%) ICC specimens positive for HPV and in 38/65 (58.5%) CIN2/3 specimens positive for HPV.

**Conclusions:**

To our knowledge this is the first report to identify HPV genotypes in cervical cancers in Zambia. A combined HPV-16/18 prevalence of 69.9% in ICC specimens suggests that current vaccines will be highly protective against cervical cancer in Zambia.

## Findings

### Background

Cervical cancer is the second most common cancer in women worldwide [1] and is the most common female malignancy referred to the University Teaching Hospital (UTH) in Lusaka [2]. Human papillomaviruses (HPVs) are responsible for over 90% of cervical cancers [3,4]. Fifteen HPV types have been associated with cervical cancer [5] and are collectively known as oncogenic or high-risk types. The two most common are HPV-16 and HPV-18, which are associated with 60–70% of cervical cancer worldwide [5]. HPV-16 and HPV-18 are the two genotypes included in currently licensed HPV vaccines (Cervarix® and Gardasil®). Cost-effectiveness modeling estimates that HPV-16/18 vaccines can decrease the lifetime risk of cervical cancer in women in Central and Eastern Africa by 36–45% [6]. However, these vaccines can only prevent cervical cancer in areas where HPV-16 and HPV-18 are primary causes. The most common genotypes isolated from cervical cancer in select African countries are HPV-16, HPV-18, HPV-35, and HPV-45 [7,8], but common genotypes in Zambia are unknown.

Studies of pre-cancer specimens in South Africa and Zambia reported a wide diversity of HPV genotypes [9,10], generating concern that current HPV vaccines might not have a large preventive effect in Zambia. However, most pre-cancerous lesions do not develop into cancer, and to determine if HPV-16 and HPV-18 cause invasive cervical cancer (ICC) requires testing ICC specimens.

To assess the impact that current vaccines can have in preventing cervical cancer in Zambia, we first compared DNA extraction techniques to determine which performed better in the Zambian context. We then estimated the combined prevalence of HPV-16 and/or HPV-18 in formalin-fixed, paraffin-embedded (FFPE) ICC and cervical intraepithelial neoplasia 2 or 3 (CIN 2/3) specimens from UTH. Our main outcomes were the percentages of specimens positive for HPV-16 and/or HPV-18.

### Methods

UTH and the adjacent Cancer Diseases Hospital in Lusaka are the primary centers for cancer diagnosis and treatment in Zambia. FFPE cervical specimens obtained during routine clinical care from 2007–2012 and stored at the UTH Pathology Department were used for this study. Unbuffered formalin is used for routine specimens at UTH, which likely leads to more degraded DNA than if buffered formalin were used [11]. Specimens were anonymized at the time of sectioning, and HIV status was unavailable. To minimize the potential for DNA contamination, gloves were worn throughout the procedure, the microtome and surrounding station were cleaned with DNAzap (Life Technologies, Carlsbad, CA, USA) or bleach before beginning work and between each specimen, and a new microtome blade and pair of gloves was used for each block. Non-HPV-containing specimens (spleen or liver) were sectioned every 15 specimens and tested; all of these controls were negative for HPV, as expected. The first and last sections of each cervical specimen block were 3 μm thick and were mounted on slides, stained with H&E, and reviewed by a pathologist at UTH to confirm the diagnosis. The middle sections (n = 3 to 9, depending on the tissue size) were 10 μm thick and were placed into 2.0 ml Screw Cap Micro Tubes (Sarstedt, Nümbrecht, Germany) and taken to the CIDRZ Central Laboratory for DNA extraction and HPV testing.

We compared two extraction techniques: heat-dependent and xylene-dependent. The heat extraction technique was adapted from Steinau et al. [12]. To each tube, 180 μl of ATL lysis buffer from a DNeasy Blood and Tissue Kit (Qiagen, Valencia, CA) was added and heated at 99°C for 30 minutes. Proteinase K (20 μl) was added, tubes were incubated at 56°C with shaking at 1,000 rpm for 16 hours, 400 μl of Buffer AL:ethanol mixture was added, and the DNA was further purified with the Qiagen kit.

The xylene extraction technique was adapted from Kocjan et al. [13]. To each specimen, 1.2 ml xylene was added, followed by vortexing and centrifugation at maximum speed for 5 minutes. The supernatant was removed, 1.6 ml of 100% ethanol was added to the pellet, followed by vortexing and centrifugation for 5 minutes. The specimen was washed two additional times and the pellet was air-dried for 15 minutes and resuspended in 180 μl of buffer ATL from a QIAamp DNA Mini Kit (Qiagen). Proteinase K (20 μl) was added, samples were incubated overnight at 56°C with shaking at 1,000 rpm, 200 μl of buffer AL was added, and DNA was further purified with the Qiagen kit. For both extraction techniques, DNA was recovered in a single elution step with 110 μl of AE solution.

Nucleic acid concentrations were determined using a Nanodrop ND-1000 spectrophotometer (Thermo Fisher Scientific, Wilmington, DE, USA), and DNA extracts were frozen at −80°C until further analysis.

Extracted DNA was tested using the Abbott RealTime High Risk HPV assay (Abbott Laboratories, Abbott Park, IL, USA), which uses real-time PCR to amplify and detect 14 high-risk genotypes [14]. RealTime also differentiates between HPV-16, HPV-18, and non-HPV16/18 genotypes (other high-risk). RealTime contains primers and probes specific for the human *β*-globin gene, to monitor DNA extraction and amplification. A cycle threshold (Ct) value of <35 was considered positive for each of the RealTime channels.

Data were entered into Microsoft Excel™ (Redmond, WA, USA), cleaned with SAS™ version 9.2 (SAS Institute Inc., Cary, NC, USA), and analyzed using SAS™ and Open Epi (www.openepi.com). p < 0.05 was considered significant.

### Results

To determine the most effective DNA extraction technique, 27 FFPE specimens (aged 3–5 years old) were extracted using the two techniques. The mean nucleic acid concentration (standard deviation) following heat extraction was 117.4 ng/μl (85.4 ng/μl), while that following xylene extraction was 38.2 ng/μl (35.9 ng/μl) (p < 0.0001, Wilcoxon signed-rank test).

The 27 FFPE specimens were tested with the RealTime assay. RealTime successfully identified HPV in the vast majority of specimens: one or more HPV was identified in 23/27 with heat extraction and 21/27 with xylene extraction (Table 1). In addition, heat extraction resulted in lower Ct values than xylene extraction (Table 1). Therefore, the remaining FFPE specimens were tested using heat extraction.

**Table 1:** **RealTime results and Ct values from DNA extracted from ICC and CIN2/3 specimens using heat or xylene treatment**

	**Heat**		**Xylene**		
**RealTime result**	**n**		**n**		
Invalid^a^	2		2		
No HPV detected	2		4		
Single HPV infection	18		19		
Dual HPV infections	4		2		
Triple HPV infections	1		0		
Total	27		27		
**Abbott Fluorophore/channel**	**n**	**Mean Ct value**	**n**	**Mean Ct value**	**p value** ^**b**^
*β*-globin internal control	25	27.7	24	30.6	**<0.001**
HPV-16	14	23.8	12	25.3	0.349
HPV-18	3	26.3	3	29.9	0.222
other HR HPV	13	28.1	9	31.0	0.156

^a^ *β*-globin internal control was either negative or had a Ct count >35.

^b^Student’s t-test.

Results represent three separate experiments of 9 specimens each (27 FFPE specimens total; 17 ICC and 10 CIN 2/3). Ct values above 35 were excluded.

Including the initial 27 specimens, 189 FFPE specimens were tested, including 114 ICC (109 squamous cell carcinoma and 5 adenocarcinoma) and 75 CIN 2/3. Of 114 ICC specimens tested, 88% were valid results (Table 2). Of the valid ICC results, HPV was identified in 93 (93%), including 78 single infections and 15 co-infections. Including specimens with multiple HPV genotypes identified, 52% of specimens were positive for HPV-16 and 25% were positive for HPV-18; together, 65/93 (70%; 95% CI: 60%–78%) were positive for HPV-16 and/or HPV-18 (Table 2). Three (60%) of the adenocarcinoma specimens had valid results; of these, HPV-16 and/or HPV-18 was identified in 2/3 (67%).

**Table 2:** **HPV results of ICC and CIN2/3 specimens tested by heat extraction and the RealTime assay**

	**ICC**		**CIN 2/3**	
	**n**	**Prevalence (95% CI)**	**n**	**Prevalence (95% CI)**
Total tested	114	--	75	--
Total with valid results^a^	100	--	69	--
Positive for any HPV^b^	93	--	65	--
HPV-16 positive	48	51.6% (41.6–61.5)	36	55.4% (43.3–66.8)
HPV-18 positive	23	24.7% (17.1–34.4)	5	7.7% (3.3–16.8)
Other HR HPV positive	38	40.9% (31.4–51.0)	43	66.2% (54.0–76.5)
HPV-16 and/or HPV-18 positive	65	69.9% (59.9–78.3)	38	58.5% (46.3–69.6)

^a^Invalid results mean the *β*-globin internal control was not reactive or had a Ct value of >35.

^b^Including specimens with multiple HPV types identified. The number of specimens that were positive for HPV was used as the denominator for prevalence estimates.

Of 75 CIN 2/3 specimens tested, 92% were valid results (Table 2) and HPV was identified in 65/69 (94%), including 48 single infections and 17 co-infections. Including specimens with multiple HPV genotypes identified, 38/65 (59%; 95% CI: 46%–70%) of specimens were positive for HPV-16 and/or HPV-18 (Table 2).

### Discussion

To our knowledge this is the first report to identify HPV genotypes in ICC in Zambia. A combined HPV-16/18 prevalence of 69.9% in ICC specimens suggests that current vaccines will be protective against cervical cancer in Zambia, and that the proportion of ICC attributable to HPV-16/18 in Zambia is similar to that in South Africa, Kenya, and the overall worldwide proportion [5].

The combined HPV-16/18 prevalence in CIN 2/3 specimens (58.5%) was lower than that in ICC specimens, but the difference was not significant (p = 0.14, two-tailed chi-square test). This is consistent with previous studies in Zambia and South Africa that reported a wide diversity of HPV types in pre-cancer samples [9,10]. The heat extraction technique performed better than the xylene extraction technique in our study, which is consistent with previous studies that report that DNA extraction without xylene is more successful than with xylene [12,15]. The Abbott RealTime assay identified HPV in over 80% of the specimens. The use of unbuffered formalin at UTH likely means that the Abbott RealTime (which has a 150 base pair amplicon) or other assays with relatively short amplicons will be most sensitive for identifying HPV in this context.

Strengths of this study include the successful development of assays to identify HPV from FFPE specimens in the Zambian context and the ability to estimate the impact of HPV vaccines on both pre-cancer and cancer. Weaknesses include the relatively small number of specimens sampled from a single referral center. However, because UTH is the primary center for cancer diagnosis and treatment in Zambia, we believe that our estimate of the prevalence of HPV-16/18 in cervical cancers is the best available for the country to date.

With an estimated 69.9% of ICC specimens at UTH containing HPV-16 and/or HPV-18, current HPV vaccines can have a substantial preventive impact on cervical cancer in Zambia. These data provide a rationale to advocate for delivery of HPV vaccines throughout Zambia. In addition, the methods described here can be used to examine the role of HPV in other cancers in Zambia, such as head and neck cancers [16,17], and the combined prevalence of HPV16/18 in ICC can be used to examine the effect of HPV vaccines, by testing FFPE specimens in the future.
